# Combining mRNA with PBS and calcium ions improves the efficiency of the transfection of mRNA into tumors

**DOI:** 10.1016/j.omtn.2024.102273

**Published:** 2024-07-17

**Authors:** Noriko Ohta, Takashi Matsuzaki, Masayoshi Nakai, Yasuhiko Tabata, Keisuke Nimura

**Affiliations:** 1 Division of Gene Therapy Science, Department of Genome Biology, Osaka University Graduate School of Medicine, Suita, Osaka 565-0871, Japan; 2 Department of DDS Pharmaceutical Development, Osaka University Graduate School of Medicine, Suita, Osaka 565-0871, Japan; 3 Department of Plastic and Reconstructive Surgery, Graduate School of Medicine, Kyoto University, Kyoto 606-8507, Japan; 4 Division of Gene Therapy Science, Gunma University Initiative for Advanced Research, Gunma University, Maebashi, Gunma 371-8511, Japan

**Keywords:** MT: Delivery Strategies, mRNA, mRNA transfection into tumors, solid tumor

## Abstract

mRNA is a promising modality for expressing a protein *in vivo*. Drug delivery systems are required for the efficient transfection of mRNA into cells. In this study, we evaluated several drug delivery systems for transfecting mRNA into tumors. A lipid nanoparticle delivered mRNA to the draining lymph nodes and liver, even by intratumoral injection. A liposome-based system did not consistently provide mRNA for different types of tumor cells. We found that PBS introduced mRNA into several tumors, and calcium ions enhanced the efficiency, particularly in male mice. The circular dichroism spectrometer suggested a structural change in mRNA in PBS. Transmission electron microscopy revealed that calcium ions promoted the formation of mRNA nanoparticles in PBS. Transfection of mRNAs coding OX40-ligand, interleukin (IL)-36γ, and IL-23 by PBS + calcium ions attenuated tumor growth. Our results indicate that combining PBS with calcium ions promotes the transfection of mRNA into tumors. These data provide information for the development of methods for transfection of mRNA for cancer therapy.

## Graphical abstract

**Figure:**
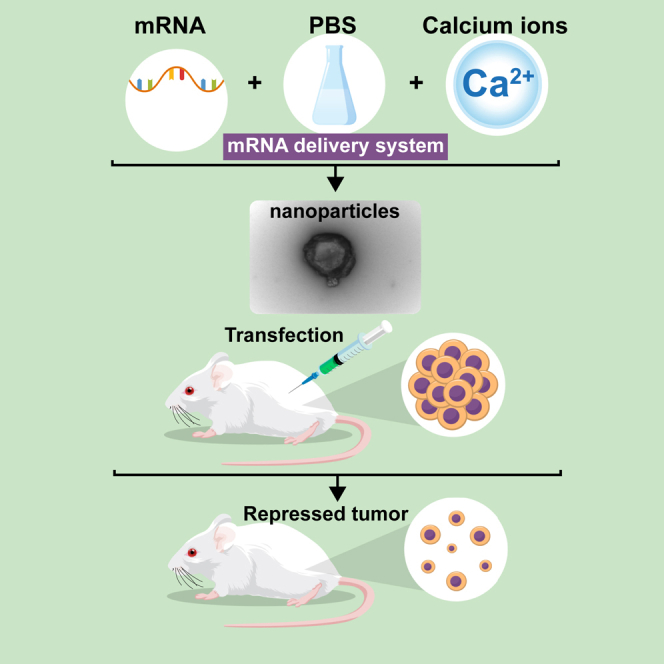

## Teaser

mRNA is a promising modality for expressing a protein *in vivo*. Nimura and colleagues’ study found that calcium ions promoted the formation of mRNA nanoparticles in PBS and introduced mRNA-coding immune-stimulating genes, attenuating tumor growth. These data provide information for the development of mRNA-based cancer therapy.

## Introduction

The mRNA lipid nanoparticle (LNP) vaccine against the coronavirus disease 2019 (COVID-19) successfully induced antibodies to the coronavirus worldwide.^1^ This success indicates that mRNA is a very promising modality for expressing proteins in the human body to cure pathogenic status. mRNA-based therapy is attractive because the mRNA sequence can be easily modified, mRNA is not integrated into the genome to prevent cancer risk, and the mRNA itself does not generate antibodies, unlike any virus-based therapy, allowing repetitive injection of mRNA.

In the cancer field, several clinical trials based on mRNA have been conducted.^2^ Two types of mRNA are used in clinical trials: mRNAs coding for neoantigen sequences to induce immunity against neoantigens^3^^,^^4^ and mRNAs coding for several cytokines to activate antitumor immunity.^5^^,^^6^ These types of mRNA may have therapeutic effects by expressing proteins coding for mRNA in some parts of the human body. An mRNA delivery system into only tumors is required for the mRNAs to alter the properties of cancer cells or directly kill them.

To develop an mRNA-based therapy, it is essential to modify mRNA, such as pseudouridine or 1-methyl-pseudouridine, to prevent recognition by the immune system^7^^,^^8^ and deliver mRNA into cells *in vivo*. LNPs have been used for mRNA delivery for COVID-19 vaccines. Some LNPs efficiently delivered mRNA to the liver and lung.^9^^,^^10^ However, generating LNPs with unique lipids in the laboratory is challenging. Instead of LNPs, several studies have reported that saline and sucrose can deliver mRNA to the heart and tumor.^11^^,^^12^ Calcium ions were used to transfect the plasmids into the cell culture system for calcium phosphate transfection. Combining serum with calcium ions forms nanoparticles in the cell culture medium; the nanoparticles are involved in the uptake of oligonucleotides.^13^ Calcium ions support the introduction of mRNA into the mouse skin.^14^ Although these studies suggest the potential of common materials as mRNA delivery systems, there is limited knowledge regarding which methods are preferable for mRNA delivery to tumors.

In the present study, we evaluated the mRNA delivery efficiency of several materials, including ultrapure water, glycerol, cationized gelatin, saline, PBS, sucrose, serum, commercial liposomes, and LNP, into several tumors using an *in vivo* imaging system (IVIS). Liposome-based *in vivo*-jetRNA introduced significant amounts of mRNA into a specific tumor. LNPs with the same composition as COMIRNATY delivered mRNA into the liver and draining lymph nodes, even by intratumoral injection. PBS consistently introduced more mRNA into several tumors than the other materials. Calcium ions enhanced the efficiency of mRNA delivery into tumors using PBS. PBS and calcium ions affected the circular dichroism (CD) spectrum of the mRNA. Calcium ions promoted the formation of nanoparticle-like structures in PBS. Furthermore, delivery of mRNAs encoding immune-stimulating molecules with PBS and calcium ions showed antitumor effects *in vivo*. Our results indicate that PBS has the ability to deliver mRNA into tumors and that calcium ions promote this activity. Our work suggests that PBS with calcium ions is a convenient method for introducing mRNA into tumors.

## Results

### The evaluation of several materials to introduce mRNA into tumors

**Figure 1:**
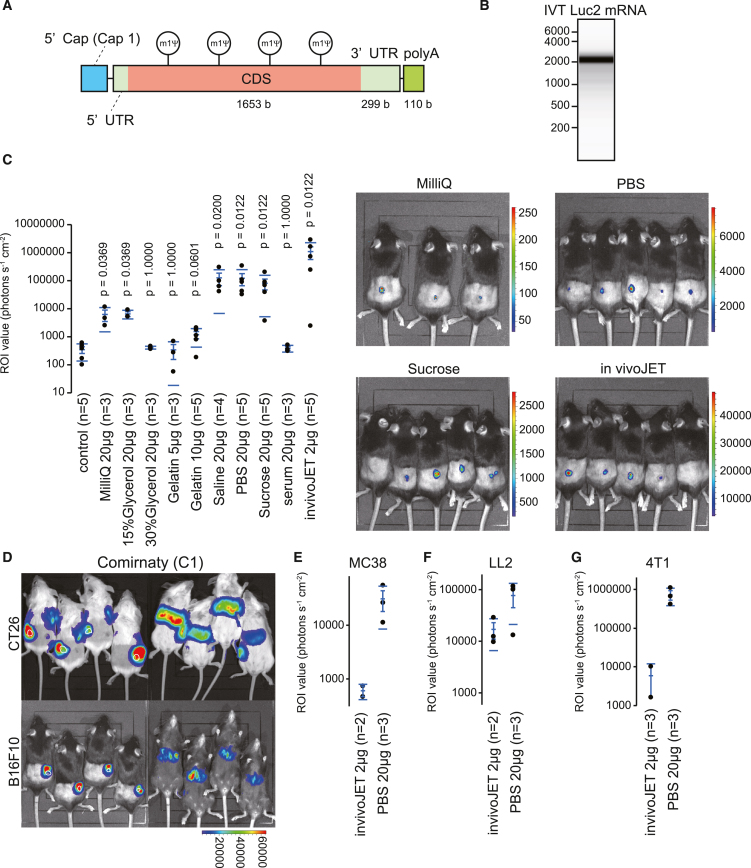
The evaluation of several materials to introduce mRNA into tumors (A) Schema of the mRNA coding luciferase 2. Luciferase 2 mRNA is generated with Cap-1 and 1-methyl-pseudouridine through *in vitro* transcription. PolyA is encoded in the template plasmid. (B) Electrophoresis of the mRNA generated through *in vitro* transcription using a Tape station. (C) Dot plot showing the region of interest (ROI) values measured using an IVIS. The number in parentheses indicates the number of tumors. We calculated *p* values using the Wilcoxon test. Right images show luciferase signals by the intra-tumoral injection of mRNA with MilliQ, PBS, sucrose, and *in vivo*-jetRNA (invivoJET). (D) Representative images of mice intratumorally injected with mRNA with COMIRNATY-composed LNP in CT26 tumor-bearing BALB/c and B16F10 tumor-bearing C57BL/6 mice. White contour indicates the location of the tumors. (E–G) Dot plot showing the ROI values in MC38 (E) or LL2 (F) tumor-bearing C57BL/6 and 4T1 (G) tumor-bearing BALB/c mice. (C, E–G) The dot plots have mean lines, mean error bars, and SD lines.

We generated the plasmid coding luciferase 2 (*luc2*) gene with the 3′ UTR of AES and mtRNR1 sequences that enhance protein expression^15^ and 110 bases polyA sequence for *in vitro* transcription using T7 (Figure 1A). The *luc2* mRNA with a cap structure and polyA was generated at the single reaction with cap-1 and 1-methyl-pseudouridine. The integrity of the synthesized mRNA was confirmed using electrophoresis (Figure 1B). Some studies have reported that saline and sucrose promote mRNA transfection into cells *in vivo*.^11^^,^^12^ Thus, we examined ultrapure water (MilliQ), glycerol, cationized gelatin,^16^^,^^17^ saline, PBS, sucrose, serum, and *in vivo*-jetRNA (Figure 1C). Luciferase signals with fewer than 10,000 regions of interest values were detected using MilliQ. While 15% glycerol allowed mRNA to enter the tumors, 30% glycerol prevented it from entering the tumors. Cationized gelatin did not promote the transfection of mRNA. Saline, PBS, and sucrose promoted the transfection of mRNA at approximately 100,000 regions of interest values. In particular, PBS introduced mRNA more consistently than saline or sucrose. The serum degraded a portion of the mRNA (Figure S1A) and prevented mRNA from entering the tumors. *In vivo*-jetRNA showed high efficiency in the transfection of mRNA, but some tumors did not express luciferase. Next, we examined COMIRNATY-composed LNP. Although intratumoral administration of LNP produced substantial signals in the tumors, we detected signals in the draining lymph nodes and liver of BALB/c and C57BL/6 mice and in the spleen of BALB/c mice (Figures 1D and S1B). We compared the efficiency of mRNA transfection between *in vivo*-jetRNA and PBS in MC38 and LL2 tumors in C57BL/6 mice and 4T1 tumors in BALB/c mice (Figures 1E–1G). We detected stronger luciferase signals with PBS than *in vivo*-jetRNA in these tumors, suggesting that the transfection efficiency of *in vivo*-jetRNA depends on tumor type. These data indicate that PBS is suitable for transducing mRNA in various tumors.

### The evaluation of types of tumors for transducing mRNA by PBS in nonobese diabetic-severe combined immunodeficiency mice

**Figure 2:**
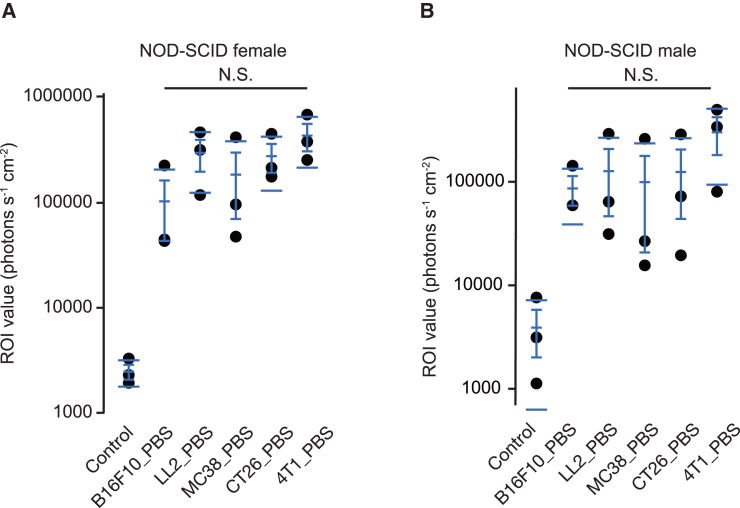
The evaluation of types of tumors for transducing mRNA using PBS in NOD-SCID mice (A and B) Dot plot with mean lines, mean error bars, and SD lines, showing the region of interest (ROI) values measured using the IVIS in the indicated tumors in NOD-SCID female (A) and male (B) mice. We calculated *p* values using the Wilcoxon test (*n* = 3). N.S., not significant.

Although we detected luciferase signals in various tumors, the signal range differed between C57BL/6 and BALB/c mice. Thus, we examined whether mouse species or tumor type caused this difference. To compare luciferase signals from various tumors in the same environment, we inoculated C57BL/6-derived B16F10, LL2, and MC38 and BALB/c-derived CT26 and 4T1 cells to immune-deficient nonobese diabetic-severe combined immunodeficiency (NOD-SCID) mice. We detected a similar range of luciferase signals in female and male NOD-SCID mice (Figures 2A and 2B). These data suggest that the efficiency of luciferase expression is equal among at least these five types of tumors and that the environment of the host animals may affect the efficiency.

### Evaluation of repetitive intratumor injection

**Figure 3:**
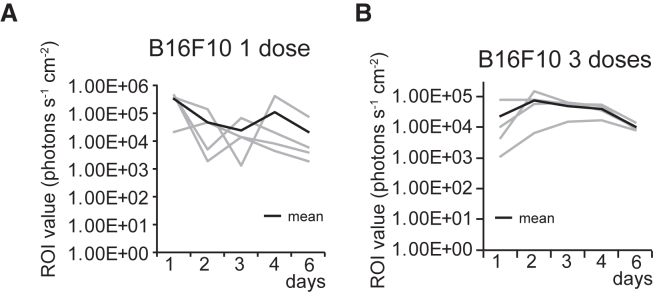
The evaluation of the repetitive intratumor injection (A and B) Line graph showing the region of interest (ROI) values measured using IVIS in B16F10 tumors in C57BL/6 mice (*n* = 4). The gray lines indicate the signals from each tumor. The black line indicates the average values from four tumors. mRNA with PBS was intratumorally injected on days 0 (A) and 0, 1, and 2 (B).

Next, we examined whether repetitive intratumoral injections of mRNA enhanced luciferase signals in tumors. One dose of mRNA with PBS expressed the luciferase activity in B16F10 tumors in C57BL/6 mice with unstable luciferase signal levels (Figure 3A). Three doses of mRNA in PBS provided stable luciferase signals in the same range (Figure 3B). These results suggest that repetitive intratumor injections have steady expression levels compared with a single dose.

### Calcium ions enhance the efficiency of luciferase expression in tumors

**Figure 4:**
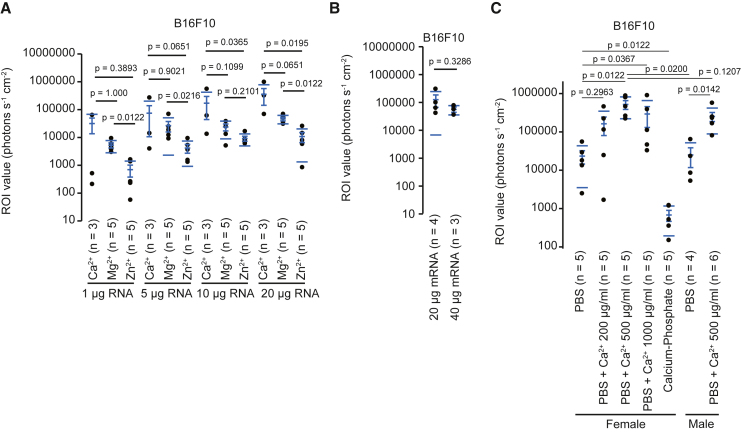
Calcium ions enhance the efficiency of luciferase expression in B16F10 tumors (A) Dot plot showing the region of interest (ROI) values in B16F10 tumors in female C57BL/6 mice. The indicated amounts of mRNA in PBS were intratumorally injected with the indicated divalent ions at 500 μg/mL. The number in parentheses indicates the number of tumors. (B) Dot plot showing the ROI values in B16F10 tumors in female C57BL/6 mice. mRNA (20 or 40 μg) was intratumorally injected into B16F10 tumors in female C57BL/6 mice. The number in parentheses indicates the number of tumors. (C) Dot plot showing the ROI values in B16F10 tumors in female and male C57BL/6 mice. mRNA (20 μg) was intratumorally injected into B16F10 tumors with the indicated buffer. The number in parentheses indicates the number of tumors. We calculated *p* values using the Wilcoxon test (A, C) and *t* test (B). (A–C) The dot plots have mean lines, mean error bars, and SD lines.

Calcium ions are involved in mRNA transfection *in vivo*.^14^ Next, we examined whether divalent ions promoted the transfection of mRNA in B16F10 tumors and whether the tumors showed luciferase signals in a dose-dependent manner (Figure 4A). With any divalent ions, 20 μg mRNA showed higher signals than 1, 5, and 10 μg. Calcium ions showed stronger signals than magnesium and zinc ions. The intratumor injection of 20 and 40 μg mRNA shows similar levels of luciferase signals (Figure 4B), suggesting that 20 μg is almost saturated for mRNA expression in B16F10 tumors. These data suggest that calcium ions may be preferable to combine with PBS to introduce mRNA into B16F10 tumors, and 20 μg is the highest amount for the intratumoral injection of mRNA.

Next, we examined the concentration of calcium ions required for mRNA transfection in B16F10 tumors (Figure 4C). In female C57BL/6 mice, 20 μg mRNA in PBS with calcium ions shows higher signals than PBS without calcium ions. Additionally, 500 μg/mL calcium ions with PBS provided steady and slightly higher signals than 200 and 1,000 μg/mL. The calcium phosphate method is used to transfect DNA *in vitro*. However, this method did not efficiently induce luciferase activity in B16F10 tumors. The combination of PBS with 500 μg/mL calcium ions also significantly enhanced the luciferase signals compared with PBS in male C57BL/6 mice. These results indicate that combining PBS with calcium ions is preferable for transfecting mRNA into B16F10 tumors in C57BL/6 mice.

### Calcium ions enhance the efficiency of luciferase expression in CT26 tumors

**Figure 5:**
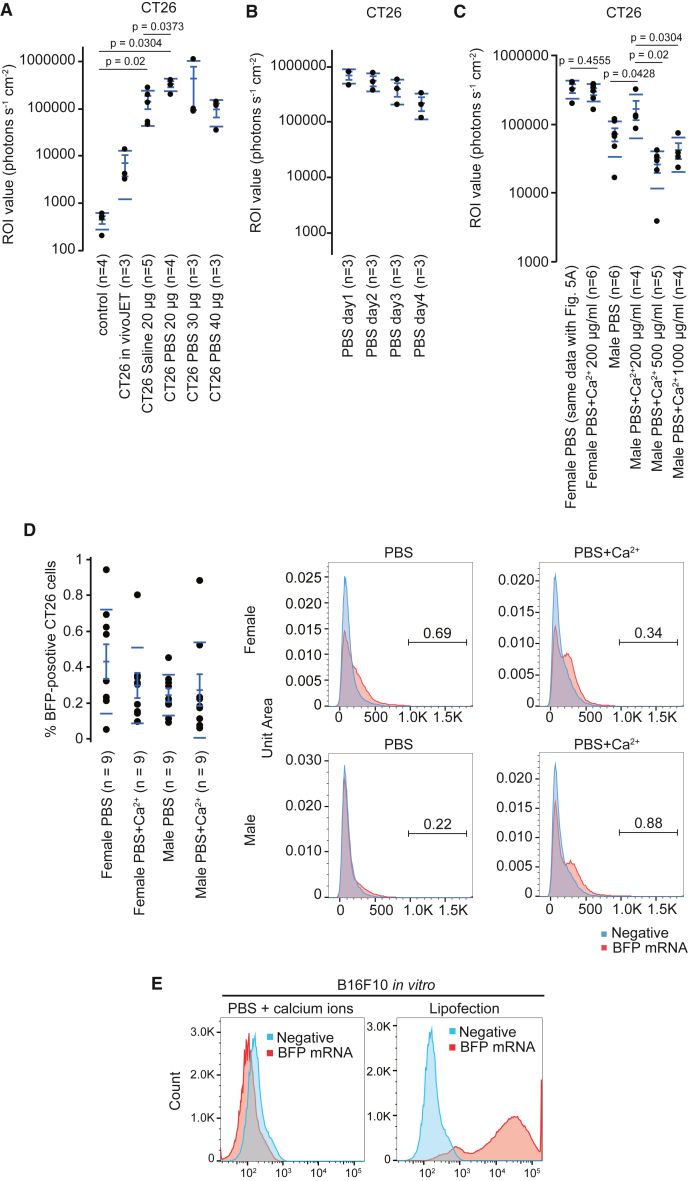
Calcium ions enhance the efficiency of luciferase expression in CT26 tumors (A) Dot plot showing the region of interest (ROI) values in CT26 tumors in female BALB/c mice. mRNA was intratumorally injected with the indicated buffer. The number in parentheses indicates the number of tumors. We calculated *p* values using the Wilcoxon test. (B) Dot plot showing the ROI values in CT26 tumors in female BALB/c mice in a time-dependent manner (*n* = 3). (C) Dot plot showing the ROI values in CT26 tumors. mRNA (20 μg) was intratumorally injected into CT26 tumors with the indicated buffer. Female PBS data is the same as CT26 PBS 20 μg in Figure 5A. The number in parentheses indicates the number of tumors. We calculated *p* values using the Wilcoxon test. (D) Dot plot showing the population of BFP-positive CT26 cells measured using FACS. mRNA (20 μg) was intratumorally injected with PBS or PBS with 200 μg/mL calcium ions. The number in parentheses indicates the number of tumors. Right histograms show the BFP-positive population in BFP mRNA-injected or control CT26 cells collected from CT26 tumors. (E) Histograms show the BFP-positive population in BFP mRNA-transfected B16F10 cells using PBS and 500 μg/mL calcium ions or Lipofectamine 2000 *in vitro*. (A–D) The dot plots have mean lines, mean error bars, and SD lines.

Next, we examined whether calcium ions also strengthened luciferase signals in CT26 tumors of BALB/c mice. PBS strengthened the luciferase signals compared with saline and *in vivo*-jetRNA (Figure 5A). The intratumor injection of 20 μg mRNA in PBS showed more stable signals than 30 and 40 μg (Figure 5A). These results indicate a superiority of 20 μg mRNA in PBS for CT26 tumors.

Luciferase signals were measured after the intratumoral injection of mRNA in PBS. We detected signals 4 days after injection, which progressively decreased in a time-dependent manner (Figure 5B).

We examined whether calcium ions strengthened the luciferase signals with PBS in the CT26 tumors of BALB/c mice (Figure 5C). We did not detect any improvement in luciferase signals in PBS with calcium ions in female BALB/c mice (Figure 5C). CT26 tumors in male mice exhibited weaker signals than those in female mice (Figure 5C). Combining PBS with 200 μg/mL, but not 500 or 1,000 μg/mL, calcium ions enhanced the luciferase signals compared with PBS in male mice (Figure 5C). We detected significant luciferase signals in the tumors but not in other tissues of mice treated with mRNA in combination with PBS and calcium ions (Figure S1C). We examined whether intratumoral injection of calcium ions changed the translation efficiency of luciferase 2 mRNA in Luc2-stably-expressed B16F10 tumors. Calcium ions did not increase the luciferase signals compared with the control (Figure S1D). These results indicate that combining PBS with 200 μg/mL strengthens the luciferase signals in CT26 tumors in male BALB/c mice.

We examined whether serum was differentially associated with mRNA depending on the species and sex. Electrophoresis of the mixture of mRNA and serum showed different patterns depending on the species and sex (Figure S1E). Because the injected mRNA is mixed with a serum component in tumors, the difference in association patterns between mRNA and serum might modify the transfection efficiency.

Next, we examined whether the combination of PBS and calcium ions increased the number of positive cells. A blue fluorescent protein (BFP)-coding mRNA was generated. We intratumorally injected the BFP mRNA into CT26 tumors of BALB/c mice (Figure 5D). We did not observe a significant difference in the population of BFP-positive cells between PBS and PBS with calcium ions in male and female mice (Figure 5D). However, we found slight peaks in the histograms of BFP-injected cells when mRNA was combined with PBS and calcium ions compared with PBS (Figure 5D). In an *in vitro* setting, combining mRNA with PBS and calcium ions did not induce BFP expression in B16F10 cells, whereas strong BFP signals were detected using Lipofectamine 2000 (Figure 5E). These data suggest that combining PBS with calcium ions slightly increases the population of weakly positive cancer cells compared with PBS alone *in vivo*.

### mRNA with PBS and calcium ions generates nanoparticle structure

**Figure 6:**
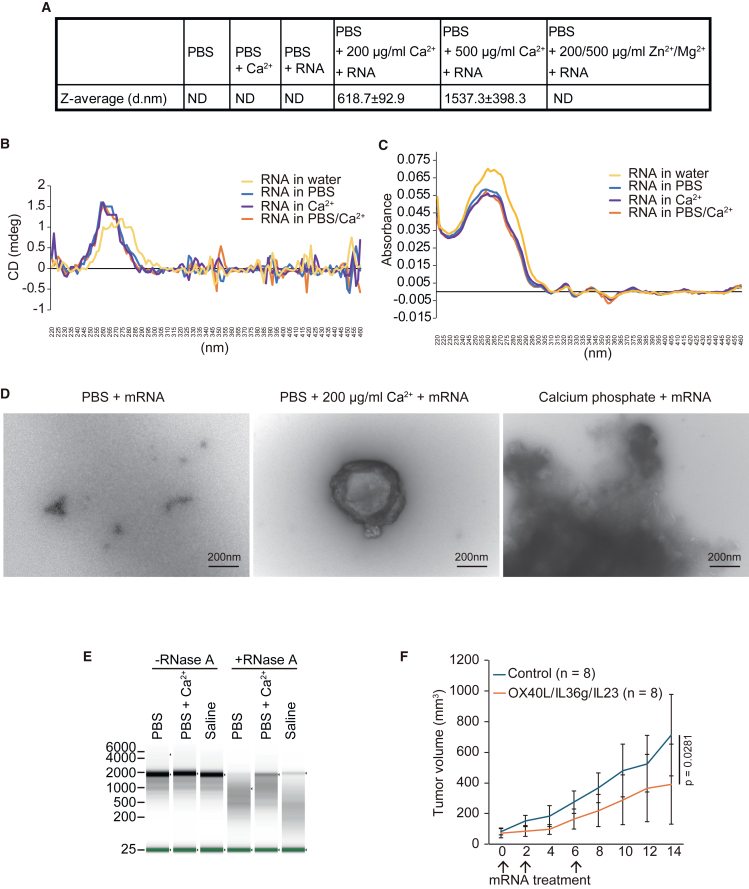
Combining PBS with calcium ions affects mRNA structure (A) Table showing the *z-*average (diameter in nm) measured using dynamic light scattering (*n* = 3). ND, not detected. (B) Line plot showing the CD spectra of mRNA in the indicated buffer. (C) Line plot showing the absorbance of mRNA in the indicated buffer. (D) Representative transmission electron microscopy images of nanoparticles in the mixture of mRNA with PBS alone, with PBS and calcium ions, or with calcium phosphate. (E) Electrophoresis of purified mRNAs after RNase A treatment. After incubation of mRNAs with the indicated buffer, mRNA was treated with RNase A. (F) Tumor growth curve of CT26 tumors. The number in parentheses indicates the number of samples. We calculated *p* values using the Wilcoxon test. Error bars show the SD.

Finally, we investigated the mechanism by which PBS and calcium ions promote the efficiency of mRNA transfection into tumors. Hori et al.^13^ reported that calcium ions and serum promote the formation of nanoparticles that enhance the uptake of oligonucleotides, but not plasmid DNA, into cells. We believe that the combination of PBS with calcium ions forms nanoparticles with mRNA. The diameters of the nanoparticles were measured using particle size analyzers (Figure 6A). We detected nanoparticles in the combination of mRNA with calcium ions and mRNA but not in PBS, PBS and calcium ions, or PBS and mRNA (Figure 6A). Additionally, 500 μg/mL calcium ions with PBS and mRNA formed larger nanoparticles than 200 μg/mL calcium ions (Figure 6A). However, zinc and magnesium ions did not promote nanoparticle formation (Figure 6A). These data suggest that the combination of mRNA with PBS and calcium ions generates nanoparticles and that this structure is beneficial for the uptake of mRNA into cancer cells.

We examined the mRNA structure in the buffer using a CD spectrometer. Folded RNA showed a higher CD spectrum at approximately 260–280 nm and a lower absorbance spectrum at approximately 250–270 nm than unfolded RNA.^18^ The right-handed A-RNA showed a CD spectrum different from that of the left-handed Z-RNA.^19^ CD spectra of mRNA showed that mRNA in water forms an unfolded structure, whereas mRNA in PBS, calcium ions, and PBS and calcium ions forms a folded structure (Figure 6B) since the CD spectra pattern was similar to that in previous reports.^18^^,^^20^ Consistent with the CD spectral pattern, the absorbance spectrum of mRNA in water was higher than that in other buffers, suggesting that mRNA in water had an unfolded structure, whereas mRNA in other buffers had a folded structure (Figure 6C).

Next, we sought to detect a nanoparticle structure of mRNA with PBS and calcium ions using transmission electron microscopy. We observed the nanoparticle structure of mRNA in the presence of PBS and calcium ions but not in the presence of PBS alone or calcium phosphate (Figure 6D). The RNase protection assay revealed that mRNA in PBS with calcium ions was more resistant to RNase A than in PBS and saline alone (Figure 6E). These results indicate that mRNA with PBS and calcium ions generates nanoparticles.

Finally, we examined whether mRNA transfection with PBS and calcium ions induced antitumor effects. The combination of interleukin (IL)-23, IL-36γ, and OX40-ligand mRNAs with LNP provides antitumor effects.^21^ Thus, we intratumorally injected mRNAs coding IL-23, IL-36γ, and OX40-ligand with PBS and calcium ions three times into CT26 tumors in BALB/c mice. The combination of the three mRNAs significantly repressed tumor growth compared with the control mRNA (*p* = 0.0281) (Figure 6F). Our data suggest that mRNA transfection with PBS and calcium ions can induce antitumor effects.

## Discussion

Delivery systems are critical for transfecting mRNA into cancer cells *in vivo*. Our study indicates that combining mRNA with PBS and calcium ions promotes the transfection of mRNA into several types of tumors *in vivo*. PBS and calcium ions change the structure of mRNA because cationic ions neutralize the negative charge of mRNA, inducing its folding.^22^ The combination of mRNA with PBS and calcium ions promotes the formation of nanoparticles. Transfection of immune-stimulating mRNAs with PBS and calcium ions induces antitumor effects. These changes in mRNA structure may affect the efficiency of mRNA transfection into cancer cells *in vivo*.

COMIRNATY-composed LNPs showed difficulty in specifically transfecting mRNA into tumors. Liposome-type *in vivo*-jetRNA introduced mRNA into a limited type of tumor. Although LNPs induce a high expression level of introduced mRNA, even by intratumoral injection, LNPs dispersed throughout the body, leading to increased mRNA expression in undesired organs, such as the liver and spleen. These properties of the LNPs may not be problematic for inducing antibodies by expressing antigens. However, LNPs with characteristics similar to those of compounds are not suitable for the specific transfection of mRNA into tumors because the dispersing expression profile of therapeutic mRNAs may cause unexpected adverse events.

We found that calcium ions promoted the transfection of mRNA into tumors more than magnesium and zinc ions, even though they are divalent ions. This result is consistent with evidence that calcium ions are classically used to transfect nucleic acids with phosphate into cells *in vitro*. Calcium ions form nanoparticles with components of serum.^13^ Nanoparticles promote the transfection of oligonucleotides but not plasmids, although oligonucleotides are not involved in nanoparticles.^13^ We found that calcium ions did not significantly change the electrophoretic profiles of mRNA in the serum. This suggests that serum promotes the uptake of oligonucleotides and that some serum components bind to mRNAs and plasmids, leading to their aggregation, which may inhibit the transfection of longer nucleic acids into cells.

PBS with calcium ions transfected mRNA into tumors *in vivo* but not into the same cancer cells *in vitro*. We believe there are two reasons for this discrepancy. One is the environment of the cancer cells. In an *in vitro* setting, cancer cells proliferate in the culture medium on a cell culture dish. mRNA nanoparticles must attach to cells and promote their uptake by cells. The culture medium may also break mRNA nanoparticles with PBS and calcium ions because mRNA nanoparticles are diluted with the culture medium. Conversely, in an *in vivo* setting, tumors have a limited amount of liquid, keeping the mRNA nanoparticle structure longer than that in an *in vitro* setting. Intratumor injection of mRNA causes pressure on cancer cells, and this pressure may help mRNA attach to cancer cells and transfect into the cells. The other is the characteristics of the cancer cells. Cancer cells have different gene expression profiles, both *in vitro* and *in vivo*, to adapt to different environments.^23^ Cancer cells *in vivo* might express a protein that takes up mRNA. We observed a limitation in the amount of mRNA expression in tumors. While increasing mRNA showed a more robust expression within 20 μg, 20 μg mRNA showed a slightly higher expression level than 30 and 40 μg. These results suggest that an mRNA uptake system exists in cancer cells. These results suggest that *in vitro* experiments may not help to explore efficient methods for delivering mRNA into cancer cells *in vivo*.

We found a difference in the efficiency of mRNA transfection between murine species and sex rather than between tumor types. mRNA in PBS induced higher luciferase signals in the tumors of female mice than in those of male mice. We did not observe increased luciferase signals in female BALB/c mice upon adding calcium ions to the mRNA in PBS. In contrast, different concentrations of calcium ions significantly increased the signals in the tumors of male BALB/c and C57BL/6 mice. In addition, 500 μg/mL calcium ions were suitable for increasing luciferase signals in C57BL/6 mice; 200 μg/mL was preferable for BALB/c mice. We thought that the serum generated the difference because serum proteins have differences in amounts and sizes depending on the murine species and sex.^24^^,^^25^ The mixture of mRNA and serum showed different patterns depending on species and sex. However, serum degraded some mRNA and inhibited their transfection into tumors, suggesting that serum promotes an association between mRNA and a serum component that may affect transfection efficiency. Human natural killer cells take up non-vesicular extracellular mRNAs using RNA-binding zinc finger proteins located on the cell membrane.^26^ This report suggests that cancer cells may also have a protein that recognizes the mRNA structure and sequence. Different concentrations of calcium ions created nanoparticles of different sizes, which may affect the efficiency of mRNA transfection into cancer cells. These data suggest that sex-related hormones may affect tumor gene expression profiles in terms of nanoparticle size.

### Limitations of the present study

Combining mRNA with PBS and calcium ions allowed the transfection of mRNA into tumors. However, the luciferase signals obtained using this method were lower than those obtained using LNPs. Although we observed the therapeutic effects of immune-stimulating mRNA injection with PBS and calcium ions, it is essential to increase the efficiency of mRNA transfection to express therapeutic proteins in patients with tumors. Since some human cells can recognize mRNA sequences in the cell membrane, optimizing mRNA sequences may be adequate to increase efficiency. The development of LNPs that precisely deliver mRNA into tumors may also improve the efficiency of mRNA transfection into tumors. In summary, we found that combining mRNA with PBS and calcium ions delivers mRNA into tumors in mice, suggesting a value of trial to examine whether the method can transfect mRNA into tumors in patients.

## Materials and methods

### Construction of plasmids

The pmRVac3-Luc2 plasmid was synthesized by VectorBuilder. pmRVac3-Luc2 plasmid contains T7 promoter with AGG sequence (TAATACGACTCACTATAAGGAGA) for integrating Cap-1 (Clean Cap Reagent AG for co-transcriptional capping of mRNA, m7G(5′) (2′OMeA)pG, *N*-7113, Trilink Biotechnologies), beta-globin leader sequence as 5′ UTR, codon-optimized *Luciferase 2*, 3′ UTR of *AES* and *mtRNR1*,^15^ poly A sequence (AAAAAAAAAAAAAAAAAAAAAAAAAAAAAAGCATATGACTAAAAAAAAAAAAAAAAAAAAAAAAAAAAAAAAAAAAAAAAAAAAAAAAAAAAAAAAAAAAAAAAAAAAAA), and SapI-recognizing sequence to linearize the plasmid without additional nucleotides at the end of polyA. pmRVac3-TagBFP was generated by swapping luciferase 2 with a TagBFP sequence. The sequences of OX40-ligand, IL-36γ, and IL-23 were amplified using PCR using cDNA from murine bone marrow cells. These sequences were introduced into the pmRVac3 plasmid.

### *In vitro* transcription

The pmRVac3 plasmid was linearized with SapI. The linearized plasmids were cleaned using a Monarch PCR & DNA clean-up kit (T1030L, New England BioLabs). mRNA was generated using the CUGA 7 *in vitro* Transcription Kit (307–13531, Nippon Gene) with N1-methylpseudouridine-5′-triphosphate (*N*-1081, Trilink Biotechnologies). After *in vitro* transcription, the template DNA was digested with DNase solution, after the purification of mRNA with PureLink RNA Mini Kit (12183018A, Thermo Fisher Scientific). The quality of the synthesized mRNA was analyzed using the 4150 Tape Station system (Agilent).

### COMIRNATY-composed LNP

We purchased (4-hydroxybutyl)azanediyl)bis(hexane-6,1-diyl)bis(2-hexyldecanoate (ALC-0315) and MPEG2000-DMG from SINOPEG (Xiamen, China). We purchased 1,2-Distearoyl-sn-glycero-3-phosphocholine (DSPC) and cholesterol from Nippon Fine Chemical Co., Ltd. (Osaka, Japan). We purchased 1,2-Dioleoyl-3-trimethylammonium-propane (DOTAP) from Lipoid GmbH. The LNP lipid composition was 50:10:39.5:1.5 at a molar ratio of ALC-0315:DSPC:cholesterol:MPEG2000-DMG. The lipids were dissolved in ethanol. The mRNA was prepared in 25 mM acetate buffer (pH 4.0). The LNP lipid solution (20 mg/mL), DOTAP solution (10 mg/mL), and mRNA solution (0.26 mg/mL) were mixed in the microfluidic mixer at a 1:0.14:3 vol and a combined final flow rate of 14.18 mL/min (3.6 mL/min LNP lipids solution, 0.5 mL/min DOTAP solution, and 10.08 mL/min mRNA solution). The LNP mixtures were then dialyzed and concentrated to approximately 0.2 mg/mL using Amicon Ultra centrifugal filters (UFC910024, 100 kD MWCO, Merck KGaA) and filtered through a 0.22-μm PES filter (16532----------K, Sartorius).

### Preparation of mRNA for the intratumor injection

For *in vivo*-jetRNA (101000013, Polyplus), 2 μg mRNA was mixed with 2 μL *in vivo*-jetRNA in 50 μL of mRNA buffer after washing mRNA with 70% ethanol twice. The mRNA was combined with PBS and ions and mixed with 10× PBS and 5 mg/mL CaCl_2_, MgCl_2_, and Zn(CH_3_COO)_2_. The mRNA combination with sucrose-citrate was at a final concentration of 0.1 g/mL sucrose and 33.3 mM citrate at pH 7.0. Phosphate-calcium comprised 20 μg mRNA with 124 mM CaCl_2_, 25 mM HEPES, pH 7.0, 140 mM NaCl, and 0.75 mM Na_2_HPO_4_. Cationic gelatin (10 μg) was mixed with 5 or 10 μg mRNA in PBS at 50 μL. Serum concentrations were measured using a BioDrop, and 200 μg serum was mixed with mRNA and 2 U/μL RNase inhibitor (F83923-1, Biosearch Technologies).

### Cell culture

B16F10 (CRL-6475), LL/2 (CRL-1642), 4T1 (CRL-2539), and CT26 (CRL-2638) cells were purchased from the American Type Culture Collection. The MC38 (ENH204) cells were purchased from KeraFast. B16F10, LL/2, and MC38 cells were cultured in DMEM (08458-45, Nacalai Tesque) co-sectioning 10% fetal bovine serum (172012, Sigma), 100 U/mL penicillin, and 100 μg/mL streptomycin (26253-84, Nacalai Tesque). We cultured 4T1 and CT26 cells in RPMI 1640 (30264-56, Nacalai Tesque) medium containing 10% fetal bovine serum with 100 U/mL penicillin and 100 μg/mL streptomycin. The cells were cultured at 37°C in a 95% humidified atmosphere with 5% CO_2_.

### *In vivo* experiments

All mouse experiments were approved by the Animal Experiments Committees of Osaka University and Gunma University. The mouse experiments were performed according to the guidelines. Moreover, 0.5 × 10^6^ cancer cells were intradermally injected into C57BL/6N, BALB/cA, or NOD.CB17-*Prkdc*^*scid*^/J (NOD-SCID) mice. mRNA was injected intratumorally 5 days after the inoculation of cancer cells.

Luciferase signals were measured using the IVIS Lumina II imaging system. D-Luciferin (XLF-1, SPI Summit Pharmaceuticals International) was dissolved in PBS at a concentration of 30 mg/mL. Then, 150 μg D-Luciferin per 1 g of mouse weight was intraperitoneally injected with 200 μL. Luciferase signals were measured after warming the mice for 3 min at 37°C under anesthesia. Luciferase signals were analyzed using Living Image 4.2.

BFP signals were measured using FACS Canto II and analyzed with FlowJo v10.9.0. Then, 20 μg of a mixture of mRNAs coding OX40-ligand, IL-36γ, and IL-23 or Luciferase 2 with PBS and 200 μg/mL calcium ions were intratumorally injected three times into CT26 tumors 5 days after inoculation in 6-week-old female BALB/c mice. Tumor size was measured using a digital caliper every other day for 14 days. Tumor volumes were calculated using the following formula: tumor volume (mm^3^) = length × (width)^2^/2.

### Measuring the *z*-average of nanoparticles and the CD and absorbance spectra

First, 5 μL of a mixture of 0.4 μg of mRNA with 200 μg/mL and 1× PBS was diluted 100 times with 200 or 500 μg/mL CaCl_2_ and 1× PBS. After 15 min of incubation at room temperature, the mixture was analyzed using a Zetasizer Nano ZS (Malvern Panalytical) and a CD-4095 Circular Dichroism Detector (Jasco Inc., Japan).

### Analyzing nanoparticles of mRNA-PBS-calcium ions using transmission electron microscopy

First, 5 μL mRNA in PBS or in PBS and 200 μg/mL CaCl_2_ was loaded onto a 400-mesh copper grid coated with a carbon support film and incubated at room temperature for 30 s. Excess liquid was removed using filter paper. The grid was washed twice with distilled water for 10 s. The grid was then negatively stained with 2% uranyl acetate droplets for 10 s. After removing excess liquid, the grid was air dried. The samples were analyzed using a transmission electron microscope (HITACHI H-7600) at an acceleration voltage of 100 kV. All the procedures were performed at the Hanaichi Ultrastructure Research Institute.

### RNase protection assay

First, 200 ng mRNA was incubated with PBS and 200 μg/mL CaCl_2_, PBS, or saline for 10 min. This mixture was incubated with 0.25 ng/μL RNase A at room temperature for 10 min. The mRNA was purified using TRIzol reagent (Thermo Fisher Scientific). The purified mRNA was analyzed using a 4150 Tape Station system (Agilent).

### Statistical analysis

Data normality was assessed using the Shapiro-Wilk test. The equal variance between the two samples was confirmed using a two-tailed f-test. Non-parametric data were analyzed using the Wilcoxon rank-sum test to compare two samples. One-way ANOVA with Tukey’s honest significant difference test was used to compare multiple groups. Error bars indicate standard deviation. Calculations were performed using JMP Pro 13 software.

## Data and code availability

The datasets generated during this study are available from the corresponding author upon reasonable request.

## Supplemental information

Document S1. Figure S1

Document S2. Article plus supplemental information
