# Supplementary material for: Combining mRNA with PBS and calcium ions improves the efficiency of the transfection of mRNA into tumors
Source: Mol Ther Nucleic Acids. 2024 Jul 17;35(3):102273. doi: 10.1016/j.omtn.2024.102273 (PMC11342175; doi:10.1016/j.omtn.2024.102273)
Supplement: Document S1. Figure S1 [file mmc1.pdf]

OMTN, Volume *35*

Supplemental information

Combining mRNA with PBS and calcium ions improves the efficiency of the transfection of mRNA into tumors

Noriko Ohta, Takashi Matsuzaki, Masayoshi Nakai, Yasuhiko Tabata, and Keisuke Nimura

Figure S1

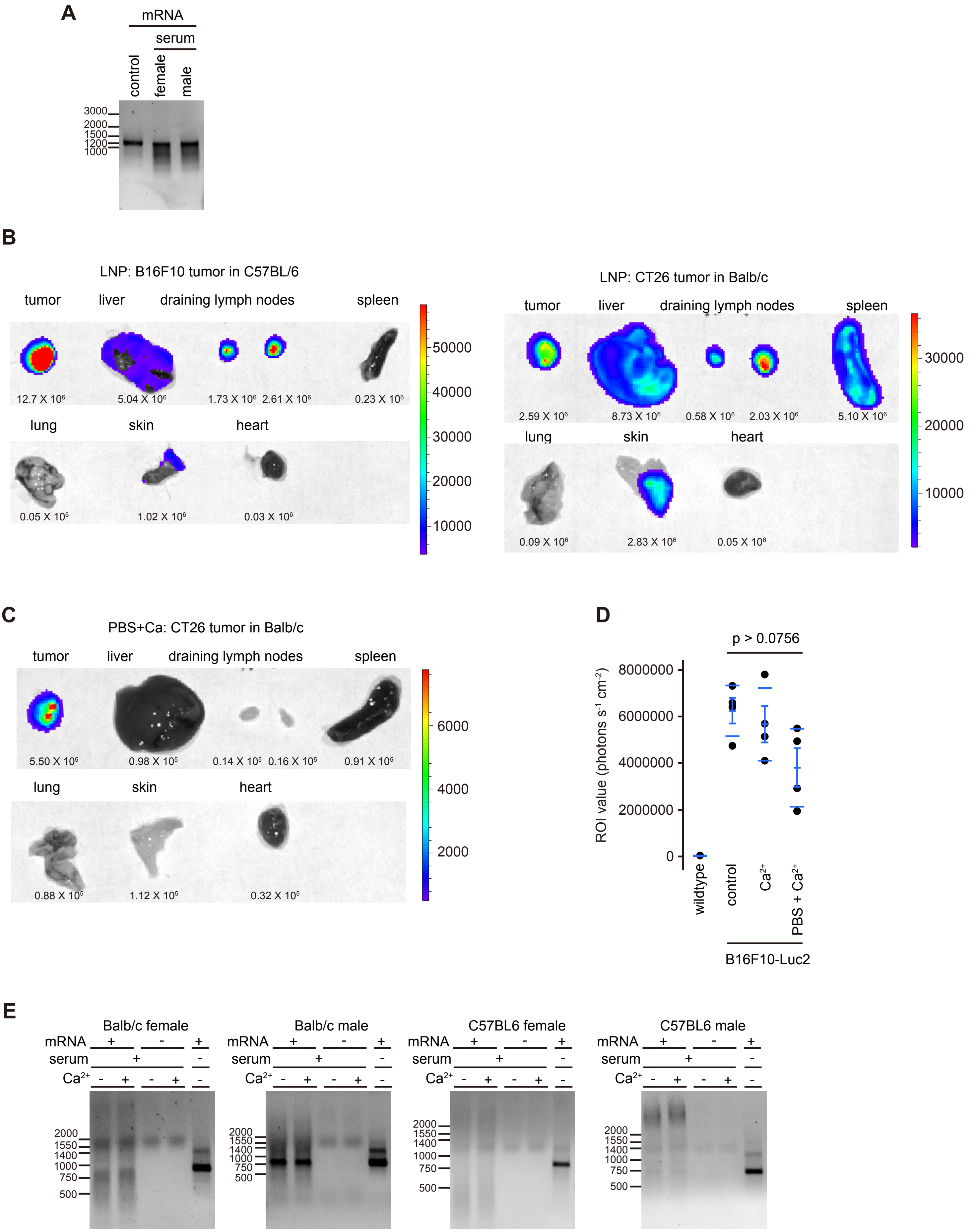

A
mRNA
serum
control
female
male
3000
2000
1500
1200
1000
B
LNP: B16F10 tumor in C57BL/6
tumor
liver
draining lymph nodes
spleen
12.7 X 10⁶
5.04 X 10⁶
1.73 X 10⁶
2.61 X 10⁶
0.23 X 10⁶
lung
skin
heart
0.05 X 10⁶
1.02 X 10⁶
0.03 X 10⁶
50000
40000
30000
20000
10000
LNP: CT26 tumor in Balb/c
2.59 X 10⁶
8.73 X 10⁶
0.58 X 10⁶
2.03 X 10⁶
5.10 X 10⁶
0.09 X 10⁶
2.83 X 10⁶
0.05 X 10⁶
30000
20000
10000
C
PBS+Ca: CT26 tumor in Balb/c
5.50 X 10⁵
0.98 X 10⁵
0.14 X 10⁵
0.16 X 10⁵
0.91 X 10⁵
0.88 X 10⁵
1.12 X 10⁵
0.32 X 10⁵
6000
4000
2000
D
p > 0.0756
ROI value (photons s⁻¹ cm⁻²)
8000000
6000000
4000000
2000000
0
wildtype
control
Ca²⁺
PBS + Ca²⁺
B16F10-Luc2
E
Balb/c female
Balb/c male
C57BL6 female
C57BL6 male
mRNA
serum
Ca²⁺
2000
1550
1400
1000
750
500

Figure S1. mRNA *in vivo* dynamics and an association of mRNA with serum. (**A**) Electrophoretic gel image of purified mRNAs after incubation with serum. (**B**–**C**) Representative IVIS images of the tissues. Numbers indicate luciferase signals for each tissue. (**D**) Dot plot of luciferase signals in B16F10 tumors stably expressing luciferase after the indicated treatments. P value was calculated using the Tukey's honest significant difference test. (n = 4) (**E**) Electrophoresis gel images of a mixture of mRNA with indicated materials. The mixtures were loaded onto gels using an SDS-free loading dye.
